# Identifying statistically significant combinatorial markers for survival analysis

**DOI:** 10.1186/s12920-018-0346-x

**Published:** 2018-04-20

**Authors:** Raissa T. Relator, Aika Terada, Jun Sese

**Affiliations:** 1 Artificial Intelligence Research Center, National Institute of Advanced Industrial Science and Technology, 2–4–7 Aomi, Koto-ku, Tokyo, 135–0064 Japan; 2 PRESTO, Japan Science and Technology Agency, 4–1–8 Honcho, Kawaguchi, Saitama, 332–0012 Japan; 3 Graduate School of Frontier Sciences, The University of Tokyo, 5–1–5, Kashiwanoha, Kashiwa, Chiba, 277–8561 Japan; 4 AIST-Tokyo Tech Real World Big-Data Computation Open Innovation Laboratory (RWBC-OIL), 2–12–1 Okayama, Meguro-ku, Tokyo, 152–8550 Japan

**Keywords:** Survival analysis, Gene marker, Multiple testing, Log-rank test, Prognosis

## Abstract

**Background:**

Survival analysis methods have been widely applied in different areas of health and medicine, spanning over varying events of interest and target diseases. They can be utilized to provide relationships between the survival time of individuals and factors of interest, rendering them useful in searching for biomarkers in diseases such as cancer. However, some disease progression can be very unpredictable because the conventional approaches have failed to consider multiple-marker interactions. An exponential increase in the number of candidate markers requires large correction factor in the multiple-testing correction and hide the significance.

**Methods:**

We address the issue of testing marker combinations that affect survival by adapting the recently developed Limitless Arity Multiple-testing Procedure (LAMP), a *p*-value correction technique for statistical tests for combination of markers. LAMP cannot handle survival data statistics, and hence we extended LAMP for the log-rank test, making it more appropriate for clinical data, with newly introduced theoretical lower bound of the *p*-value.

**Results:**

We applied the proposed method to gene combination detection for cancer and obtained gene interactions with statistically significant log-rank *p*-values. Gene combinations with orders of up to 32 genes were detected by our algorithm, and effects of some genes in these combinations are also supported by existing literature.

**Conclusion:**

The novel approach for detecting prognostic markers presented here can identify statistically significant markers with no limitations on the order of interaction. Furthermore, it can be applied to different types of genomic data, provided that binarization is possible.

## Background

Survival analysis is generally used in studies whose primary interest is the time of occurrence of an event. For instance, one may be interested in the time from first treatment of leukemia patients to time of remission, the time from first heart attack until death, or the time from being cancer-free to time of recurrence. Unlike ordinary regression models, survival analysis methods can incorporate censorship and time information which are usually present in clinical data. They can also be used to estimate survival, or the probability of surviving up to a certain time, and hazard, or the instantaneous rate of occurrence of the event. In addition, they can be utilized to describe the effects of important factors on the survival of the individual, such as age, gender, or treatment. In a similar manner, we can take advantage of these methods to help identify significant biomarkers for survival.

Prognostic biomarkers for diseases like cancer are commonly identified using genomic data such as genome-wide expression profiles [1–6]. Recent technologies have led to the increase in the number of biomarkers, and discovery of combinatorial effects of markers has been anticipated, especially in complex diseases where gene interactions may play important roles in regulatory pathways. Various algorithms and techniques have already been developed for marker detection, with strategies ranging from variable selection to Cox score ranking [2] and log-rank test. However, owing to the high dimension of data leading to combinatorial explosion, most existing methods can only exhaustively inspect individual candidate markers, failing to consider high order interactions. In addition, multiple hypothesis testing has also complicated the evaluation of statistical significance of detected markers, even in individual inspections, as the large correction factor limits novel discovery from data.

**Fig. 1:**
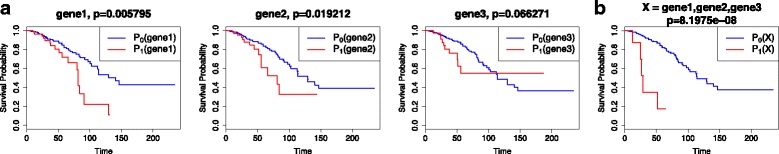
Example of a statistically significant combination marker *X* with some non-significant gene components. **a** *P*_1_(gene) denotes samples with over-expression of the corresponding gene and *P*_0_(gene) denotes samples with no over-expression of the gene. Under *α*=0.05, gene3 is not significant, and under *α*=0.01, only gene 1 will be significant. **b** The gene combination *X* is comprised of the three genes in (**a**). *P*_1_(*X*) denotes the samples with over-expression of all 3 genes in *X* and *P*_0_(*X*) represents the samples with no over-expression in at least one of the 3 genes. The combination of the three genes results to a marker with significantly low *p*-value even if not all three are evaluated as significant. With a conventional filtering approach, this combination marker may never be detected

If only single markers or pair markers were considered for statistical assessment, it would be computationally feasible to exhaustively test each candidate. But given the size of standard genomic data and that the size of the combination is arbitrary, the number of tests can be exceedingly large, leading conventional methods for identifying prognostic markers to perform statistical assessment on individual genes or individual SNPs only. This leaves several prospective markers, such as those of high order combinatorial interactions, untested for significant effects. Other approaches try to perform a screening step to narrow down candidates involved in combinations. For example, a subset of the original set of markers may be retained based on their individual statistical significance after performing some initial evaluation. Then, higher order candidate combination markers are generated by considering interactions of the markers retained in this subset and assessed for significant associations. Wang et al. adopted this strategy by restricting the set to the top significantly differentially expressed genes first before generating and selecting candidate combinations using a robust likelihood-based procedure [5]. In a similar manner, Li et al. also initially screened genes by performing survival analysis and retaining those whose expressions are correlated with patient survival for further analyses [1]. The work of van’t Veer et al. implemented the same technique and used the correlation coefficient values to select significantly associated genes and generated marker combination for accurate prognosis classification using the ranked coefficients [6]. Though feasible, screening strategies disregard the possibility of significant combinations of individual markers with insignificant effects, as illustrated in Fig. 1. In the illustration, suppose *X* in Fig. 1b is a combination of three genes: gene1, gene2, and gene3 in Fig. 1a. Depending on the correction factor used, gene 1, 2, or 3 may not be identified as significant, therefore *X* may not be discovered as a candidate marker. All the while, *X* shows noteworthy effect on the survival of individuals in Fig. 1b.

To overcome the dilemma occurring in statistical assessment of multiple hypotheses, the Limitless Arity Multiple-testing Procedure (LAMP) was proposed by Terada et al. for finding significant motif combinations that regulate gene expressions [7]. Using frequent pattern mining [8], the method can enumerate all combinations of transcription factors that are statistically significantly associated with the up-regulation of genes. Furthermore, the probability of at least one false discovery occurring is guaranteed to be less than the predefined threshold *α*, usually 0.05 or 0.01 in value, by excluding infrequent combinations that will never be significant, and hence do not contribute to the family-wise error rate (FWER), or the probability of making at least one false discovery [9]. However, the theory of LAMP is only valid for Fisher’s exact test, chi-square test and Mann Whitney U test, and is not directly applicable to survival analysis.

In this research, we propose an extension of LAMP for log-rank test to detect prognostic gene combinations. Log-rank test is commonly used for differentiating chances of survival between groups. It can also be interpreted as a time-stratified Cochran-Mantel-Haenszel test (CMH) [10]. The CMH test is used to test for association between a binary predictor, such as treatment, and a binary outcome, like case or control, while taking stratification into consideration. For log-rank, we can assume that the binary predictor is given by the categories of the two populations, such as presence of marker, and the binary outcome is the occurrence of the event at the given failure time. With this setting, we can test for association between the failure of samples and the grouping of samples. Most existing methods support their results with *p*-values computed using log-rank, like in the work of duVerle et al. [11]. To find marker combinations, their method treats combinations as covariates and integrates penalized Cox regression analysis with significant pattern mining to find combinatorial interactions. Their algorithm runs for several iterations to find candidate combinations with significant likelihood ratio test *p*-value, and later test them using the log-rank *p*-value. Statistical significance of their detected combinations is not necessarily guaranteed. On the other hand, our approach directly exploits the log-rank *p*-value to identify meaningful individual markers and multiple-marker interactions. By modifying LAMP, the procedure becomes more suitable for survival data, which generally involves censored information, while enabling us to identify high order combinations without dealing with issues raised by test multiplicity. Similar to [11], our approach sets no limit on the order of the detected interactions. But unlike them, it does not require training of algorithm that causes possible overfitting of data.

We applied our algorithm to datasets of mRNA expression profiles from The Cancer Genome Atlas (TCGA). Cancer is a complex disease whose course and prognosis is highly variable, and some cancer types cause more deaths than others, such as lung, liver, stomach and breast cancers.Therefore, treatment options differ for each individual, and it has been essential to establish prognosis of patients. Aside from early detection before the spread of the disease being crucial, prognostic and predictive markers have also become highly relevant in personalizing medical care and improving the quality of treatment. Our method identified combinatorial interactions with orders of up to 32 genes, and existing studies can confirm the effects of some these genes included in these combinations. Additionally, the method presented here is not restricted to gene expression data, but can also be applied to other types of genomic data such as copy number variations, or single-nucleotide polymorphisms, as long as binarization of values can be performed. This makes our strategy more flexible than other data-defined methods for marker identification.

## Methods

### Overview

In this study, we will focus on the following problem setting. Suppose we have a survival dataset composed of a set of markers $\mathcal {G} = \{g_{i}\}_{i=1}^{M}$ and a set of individuals $\{s_{\ell }\}_{\ell =1}^{N}$ with their corresponding survival times $\{\tau _{\ell }\}_{\ell =1}^{N}$. Here, we will assume that there is only one level of expression of the marker *i* for each sample *ℓ*, i.e. *g*_*i*_(*s*_*ℓ*_)∈{0,1}{*i*=1,2,…,*M*},{*ℓ*=1,2,…,*N*}. For example, *g*_*i*_ may represent a single gene, highly expressed genes are denoted by 1 and not highly expressed genes are denoted by 0. When *g*_*i*_ is assumed as a SNP, 1 and 0 mean minor homozygous SNP and non minor homozygous SNP, respectively. In addition, let $\{y(s_{\ell })\}_{\ell =1}^{N}$ be the corresponding labels of each individual such that *y*(*s*_*ℓ*_)=1 if the event of interest occurred for the individual, which we refer to as a failure or failed sample, and *y*(*s*_*ℓ*_)=0 if the information on the sample is censored. Let *X* be a pattern of *m* markers $\{g_{i}\}_{i=1}^{m}$ drawn from the powerset of $\mathcal {G}$, and $\{t_{j}\}_{j=1}^{K} \subseteq \{\tau _{\ell }\}_{\ell =1}^{N}$ be the unique failure times, that is, there is at least one sample *s*_*ℓ*_ such that *τ*_*ℓ*_=*t*_*j*_ and *y*(*s*_*ℓ*_)=1. Then for any failure time *t*_*j*_, we can also subdivide the *N* individuals into two groups: *P*_1_={*s*_*ℓ*_|*g*_*i*_(*s*_*ℓ*_)=1 ∀*g*_*i*_∈*X* and *τ*_*ℓ*_≥*t*_*j*_} or the set of samples containing pattern *X* who survived to at least until *t*_*j*_, and $P_{0}=\overline {P_{1}}=\{s_{\ell }|\ \exists g_{i}\in X, g_{i}(s_{\ell })=0~ \text {and} ~\tau _{\ell }\geq t_{j} \}$ or the set of samples not containing *X* who also survived to at least until *t*_*j*_. Our goal is to detect combinations *X* such that survival times of individuals from the two groups *P*_1_ and *P*_0_ are statistically significantly different, while taking censored information into account. Thus, we can say that *X* is associated to survival, making it a promising candidate marker.

A statistical test for survival analysis, such as the log-rank test, is useful to evaluate statistical significance of a combination like *X*. But to use it to exhaustively investigate the effects of combination markers, statistical assessment must be performed for every possible combination, i.e., 2^*M*^−1 statistical tests are performed. Such approach does not only cause computational complexity problems, but also yields a serious number of false discoveries. To overcome these problems, we present an algorithm for finding combinatorial interactions significantly associated with the survival of individuals while controlling FWER and correcting for multiple hypotheses. To achieve this goal, the proposed method integrated the statistical evaluation capability of the log-rank test with the multiple testing correction power of LAMP.

### The Log-rank Test

The log-rank test is used to determine statistical difference in the time-to-event for any given time between the two populations. For example, one might be interested in the time before death between treatment and placebo for a complex disease in a clinical trial. The test assumes that occurrence of event is not dependent on censoring, and that event probabilities are unaffected by the start times of the individuals in the study [12].

Given a combination of markers $X \subseteq \mathcal {G}$, we construct sequential contingency tables to calculate the *p*-value with the log-rank test. Table 1 shows a contingency table for time *t*_*j*_, where *t*_1_<⋯<*t*_*j*_<⋯<*t*_*K*_ are the ordered failure times. Let *Y*_*j*_ and *n*_*j*_ be the numbers of individuals satisfying *τ*_*ℓ*_≥*t*_*j*_ and failed individuals satisfying *τ*_*ℓ*_=*t*_*j*_, respectively. And let *λ*_*j*_ be the number of individuals in *P*_1_ at *t*_*j*_ and *n*_1*j*_ be the number of individuals in *P*_1_ such that *τ*_*ℓ*_=*t*_*j*_ and *y*=1.

**Table 1:** Contingency table at *j*th failure time *t*_*j*_

	*y*=1 at *t*_*j*_	*y*≠1 at *t*_*j*_, *τ*_*ℓ*_≥*t*_*j*_	Row totals
*P*_1_	*n*_1*j*_	*λ*_*j*_−*n*_1*j*_	*λ*_*j*_
*P*_0_	*n*_*j*_−*n*_1*j*_	*Y*_*j*_−*λ*_*j*_−*n*_*j*_+*n*_1*j*_	*Y*_*j*_−*λ*_*j*_
Col totals	*n*_*j*_	*Y*_*j*_−*n*_*j*_	*Y*_*j*_

For the *j*th failure time, the observed number of failures in *P*_1_ is given by *n*_1*j*_ and the expected number of failures equal to

$$E_{j}=n_{j}\frac{\lambda_{j}}{Y_{j}}. $$

The log-rank statistic *Z* measures the ratio between the summed deviation between the observed failures and the expected failures for each failure time, normalized by the square root of the summed variance for each failure time:

$$\begin{array}{@{}rcl@{}} Z & = & \frac{\sum_{j=1}^{K}n_{1j}-E_{j}}{\sqrt{\sum_{j=1}^{K}V_{j}}}, \end{array} $$

where the variance at *t*_*j*_ is given by

$$V_{j}=\frac{\lambda_{j}(Y_{j}-\lambda_{j}-n_{j}+n_{1j})n_{j}(Y_{j}-n_{j})}{Y_{j}^{2}(Y_{j}-1)}. $$

The test statistic *Z*^2^ has a chi-square distribution with 1 degree of freedom, which can be used for statistical assessment of survival curves of the two groups *P*_1_ and *P*_0_ via chi-square test. That being the case, we can explicitly use the test to find statistically significant markers that influence the survival of the individuals.

### LAMP

Multiple hypothesis testing is one of the many challenges in finding significantly associated markers in disease survival and disease incidence. Several methods have been proposed to address this problem, with Bonferroni correction as one of the most highly utilized approaches. This is easily performed by dividing the predetermined significance level *α* (generally 0.05 or 0.01) by the total number of hypotheses to be tested, *k*, to obtain the adjusted *p*-value threshold *δ*. However, this method is known to be conservative. Especially when considering higher order interactions, the number of tests easily increases exponentially, causing the adjusted threshold to be very small and discouraging new findings from the data.

As a workaround on the drawback of Bonferroni correction, one strategy is to determine which tests are “testable” and “untestable” [9]. This is the technique used by Terada et al. for controlling the FWER in statistical tests such as Fisher’s exact test and chi-squared test [7]. For a test with contingency table marginals given by *n*_1_,*λ*,*N*−*n*_1_ and *N*−*λ*, where *n*_1_ is the total number of samples with label 1, and *λ* is the the total number of occurrences of a pattern of markers *X*, the minimum raw *p*-value is obtained when the table is most biased and so cannot be less than

1 $$ f(\lambda)=\frac{\binom{n_{1}}{\lambda}}{\binom{N}{\lambda}}.   $$

Therefore, if for some *λ*,*f*(*λ*) is greater than the adjusted *p*-value *δ*, then the corresponding pattern of markers can never be significant and is therefore untestable.

To apply the above method to finding high order combinations of transcription factors regulating gene expression, Terada et al. used the linear-time closed itemset miner (LCM) [8]. LCM can enumerate patterns whose frequency of appearance in the data is at least equal to *λ*. When the minimum *p*-value bound *f*(*λ*) is used with LCM, significant patterns can be identified by using the anti-monotonic property of *f*. The LAMP algorithm [7, 13] is outlined in Algorithm 1. First, *λ* is set to the minimum between the maximum frequency over all patterns *X* in the data and the total number of positive labels *n*_1_ in the data. Then, the LCM algorithm is called to list all patterns with frequencies no less than *λ*, with *k* equal to the order of this set. If *f*(*λ*−1)≤*α*/*k* using Eq. 1, then *λ* is decremented by 1, and LCM is called again to find all corresponding patterns and compute for the new *k*. The last two steps are repeated until *f*(*λ*−1)<*α*/*k* or until *λ*=2. The algorithm outputs the optimal *λ*^∗^, an exhaustive list of the testable patterns, and the total number of testable patterns corresponding to *λ*^∗^.



### LAMP for survival analysis

LAMP can be used to find associations using statistical tests, but it cannot be directly applied to survival data. Therefore, it is necessary to extend the algorithm to incorporate censorship and time information. An attractive point of the approach is that it is easily applicable to methods provided we can find a non-zero bounding function for the minimum *p*-value that is monotonically decreasing [7]. To this end, we define such function for the log-rank test.

#### *Proposition 1.*

Let *λ*, *n*_1*j*_, *n*_*j*_, and *Y*_*j*_ be contingency table values and marginals previously defined. The minimum *p*-value of the log-rank test is bounded below by the monotonically decreasing function

2 $$\begin{array}{@{}rcl@{}}  f(\lambda) & = & \prod\limits_{j=1}^{K}f_{j}(\lambda), \\ \text{where}\; f_{j}(\lambda) & = &\left\{\begin{array}{ll} \binom{n_{j}}{\lambda}\Big/\binom{Y_{j}}{\lambda} &,\ \lambda\leq n_{j}\\ 1\Big/\binom{Y_{j}}{n_{1j}} &,\ \lambda>n_{j}. \end{array}\right.  \end{array} $$

#### *Proof*

Let $\chi ^{2}_{\text {LR}}$ be the the log-rank chi-square statistic and *p*_*j*_ be the corresponding *p*-value for the 2×2 contingency table at the *j*th failure time *t*_*j*_, *j*=1,2,…,*K*. We consider Fisher’s method and the unified statistic given by

$$\chi^{2}_{\mathrm{u}}(2K) \sim -2\sum_{j=1}^{K}\ln p_{j}. $$

This statistic is sensitive to small values of *p*_*j*_ and tends to be large if at least one null hypothesis *H*_*j*_ is not true. Thus, for values at the right tail of the distribution, given that the degree of freedom 2*K* of $\chi _{\mathrm {u}}^{2}$ is greater than the degree of freedom of $\chi _{\text {LR}}^{2}$ (df =1) but $\chi _{\mathrm {u}}^{2}$ is large enough to also reject the null hypothesis as $\chi _{\text {LR}}^{2}$, then $\chi _{\text {LR}}^{2}<\chi _{\mathrm {u}}^{2}$. In a similar manner, this also holds when all null hypotheses are true. If $\chi _{\text {LR}}^{2}$ is small and null hypothesis is true, then *p*_*j*_’s also tend to be large. However, the log-rank *p*-value *p*_LR_ is only large for extreme values of $\chi _{\text {LR}}^{2}$. Therefore to achieve comparable probability as log-rank at a higher degree of freedom, the combined statistic $\chi _{\mathrm {u}}^{2}$ is still greater than $\chi _{\text {LR}}^{2}$. Moreover, for nontrivial values of *K*, $\chi _{\text {LR}}^{2}\ll \chi _{\mathrm {u}}^{2}$, so the corresponding *p*-values $p_{\text {LR}}=p\left (\chi _{\text {LR}}^{2}(1)\right)>p\left (\chi _{\mathrm {u}}^{2}(2)\right)$, i.e., if $p\left (\chi _{\text {LR}}^{2}(1)\right)>p\left (\chi _{\mathrm {u}}^{2}(1)\right)$, the $\chi _{\mathrm {u}}^{2}$ is sufficiently large such that inequality still holds when its df is increased by 1. We choose df =2 to take advantage of the equivalent distribution of *χ*^2^(2), and rewrite

$$p_{\text{LR}}>p\left(\chi_{\mathrm{u}}^{2}(2)\right)=\exp\Bigg(\ln\Bigg(\prod_{j=1}^{K}p_{j}\Bigg)\Bigg)=\prod_{j=1}^{K}p_{j}. $$

Thus, we can bound the log-rank *p*-value by the product of respective *p*-values of each table.

Note that as the failure time *t*_*j*_ becomes longer, entries of the *j*th contingency table and sample size becomes smaller. Therefore, it is preferable to use Fisher’s exact test to compute for the corresponding *p*-value of the table instead of chi-square test. Under the null hypothesis, the probability of generating a contingency table such as in Table 1 at each failure time is equal to the probability for a single 2×2 table in Fisher’s exact test [14]. The corresponding *p*-value of the table at *t*_*j*_ is given by

$$f_{j}(\lambda_{j})=\frac{\binom{n_{j}}{n_{1j}}\binom{Y_{j}-n_{j}}{\lambda_{j}-n_{1j}}}{\binom{Y_{j}}{\lambda_{j}}}. $$

Moreover, this achieves its minimum when the table is most biased [7]. Therefore, $f_{j}(\lambda _{j})=\binom {n_{j}}{\lambda _{j}}\Big /\binom {Y_{j}}{\lambda _{j}}$ if *λ*_*j*_≤*n*_*j*_ and $f_{j}(\lambda _{j})=1\Big /\binom {Y_{j}}{n_{1j}}$ if *λ*_*j*_>*n*_*j*_. Fixing *λ*_*j*_=*λ* for all *j*, we get the bounding function defined in Eq. 2.

To show that *f* is monotonically decreasing, observe that when *λ*≤*n*_*j*_:

$$\begin{array}{@{}rcl@{}} f_{j}(\lambda + 1) &=& \frac{\binom{n_{j}}{ \lambda + 1}}{\binom{Y_{j}}{\lambda + 1}} = \frac{n_{j} - \lambda}{Y_{j} - \lambda}\frac{\binom{n_{j}}{\lambda}}{\binom{Y_{j}}{\lambda}} \\ &=& \frac{n_{j} - \lambda}{Y_{j} - \lambda} f_{j}(\lambda)  \\ \end{array} $$

And since *n*_*j*_≤*Y*_*j*_, then (*n*_*j*_−*λ*)/(*Y*_*j*_−*λ*)≤1. On the other hand, when *λ*>*n*_*j*_, *f*_*j*_(*λ*) is independent of *λ*. Therefore, *f*_*j*_(*λ*) decreases with respect to *λ*, and the conclusion follows. □

### Algorithm

To find statistically significant interactions using log-rank test, we implemented the following algorithm tailored from the original LAMP algorithm [13]. Briefly, the differences of the two algorithms are the initialization of *λ* and the computation of the minimum *p*-value bound.

Similar to LAMP, *λ* is initially set to the maximum frequency over all patterns *X* in the data. If this value is larger than the minimum number of samples at risk *Y*_*j*_ over all failure times, *λ* is set to this value in line 2. Lines 4–5 call the LCM algorithm while decreasing the value of *λ* by 1 for each iteration to find all patterns *X* whose number of occurrences is at least *λ*, until the value *f*(*λ*−1)≤*α*/*k*, *k* equal to the number of such patterns *X*. The value of *f* is computed using the bound defined in Eq. 2. The *p*-value for the corresponding *λ* is computed in each failure time, and the product across all failure times is obtained. When the condition in line 5 is not met, or if the current *λ* is already equal to 2, then algorithm finally outputs the optimal value of *λ*, an exhaustive list of all testable patterns corresponding to this value, and the total number of these patterns, *k*.



### Data

To test our approach, the algorithm was applied to two publicly available datasets with clinical data from The Cancer Genome Atlas (TCGA) Database: samples from breast invasive carcinoma (TCGA-BRCA) [15] data and samples from ovarian serous cystadenocarcinoma (TCGA-OV) [16] data. The TCGA-BRCA data contains 14688 mRNA gene expression profiles from 526 samples, while the TCGA-OV data has from 17578 mRNA gene expression profiles from 485 samples. The event of interest is death of the individual, with overall survival time (in months, from time of enrollment in study until death) given. TCGA-BRCA contains 419 distinct survival times, with 65 distinct failure times, while TCGA-OV has 433 unique survival times and 252 unique failure times. The *z*-scores of median-centered per gene data were provided, and we used this to binarize the expression values such that *z*-scores greater than 2 are classified as highly expressed. The average number of highly expressed samples per gene was around 21 samples for both data. To finish the computations within three days, we opted to divide the data into sets with 250 genes per set (directly as given in order of the data; last set may have < 250 genes) and implemented the algorithm per set. This yielded 59 sets for the TCGA-BRCA data and 79 sets for the TCGA-OV data. We aggregated the results for all experiments and used the total correction factor for all analyses as the significance threshold correction factor. We filtered the significant gene interactions detected by our algorithm by selecting those whose raw *p*-value multiplied by the total correction factor is still less than the threshold *α*, set here to 0.05. We performed all our experiments in a machine with two Intel Xeon E5-2650 v3 (2.30GHz) processors with 128GB memory.

## Results and discussion

### Analysis of TCGA breast invasive carcinoma data

We obtained a total of 9634 statistically significant combinations from TCGA-BRCA, and the average correction factor per analysis is 9428. We used the total correction factor *k*=556284 to retain statistically significant combinations across all analyses, reducing the number of significant interactions to 5836 with the largest size of gene combinations is 32. Due to some unexpected bias that may be presented when detected significant markers only affect one or very few samples in the whole the data, we sorted the combinations in decreasing number of occurrences of the marker combination. Table 2 gives the first 5 of the sorted interactions found to be statistically significantly associated to breast cancer prognosis by our method.

**Table 2:** Significant markers with the most number of occurrences in the breast invasive carcinoma data

Gene combination	Adj. Log-rank *p*-value	No. of occurrences
C1orf55,TIMM17A	0.0185	23
TIMM17A,OTUD6B	0.0025	14
MRPS14,ZNF707,TTC35	0.0002	12
C1orf55,TIMM17A,OTUD6B	0.0007	11
C8orf38,ZC3H11A	0.0015	11

The combinations yielded by our analysis involve genes that have been previously implicated in disease incidence or associated with disease prognosis. These include the PIK3CA gene (part of a combination of 28 genes, raw *p*=1.9545*e*−09, adjusted *p*=0.00109), which is one of the three genes whose occurrence of somatic mutations are greater than 10% among all breast cancers [15], and BRCA2 (first combination has size 22, raw *p*=1.9545*e*−09, adj. *p*=0.00109; second combination has size 8, raw *p*=3.3648*e*−08, adj. *p*=0.01871). From the table, the frequently appearing TIMM17A gene is a known breast cancer marker [17], and have been previously shown to affect the aggressiveness of tumor cells in breast cancer [18, 19]. High expressions of this gene have been linked to the more progressive type of the disease, resulting to unfavorable survival outcomes for affected patients.

The other genes, while not directly associated to breast cancer survival, have also been studied for associations with breast cancer, other cancer types, or cancer risk. For instance, C1orf55 (SDE2) gene in the first and fourth combination in Table 2 has been recently shown to help cells in replication stress relief [20], and replication stress is known to correlate with the formation of tumors or tumorigenesis [21]. Another example is the OTUD6B gene in the second and fourth combinations, which is a gene belonging to a subfamily of ovarian tumor domain, and a potential biomarker for non-small cell lung cancer [22]. Additionally, gene expression of ZNF703 in combination three has been shown to activate gene expression that lead to increase in cancer stem cells, which promote tumorigenesis, in breast cancer [23]. From the aggregated results in the analyses, we obtained a total of 5930 unique genes included in the combinations.

**Fig. 2:**
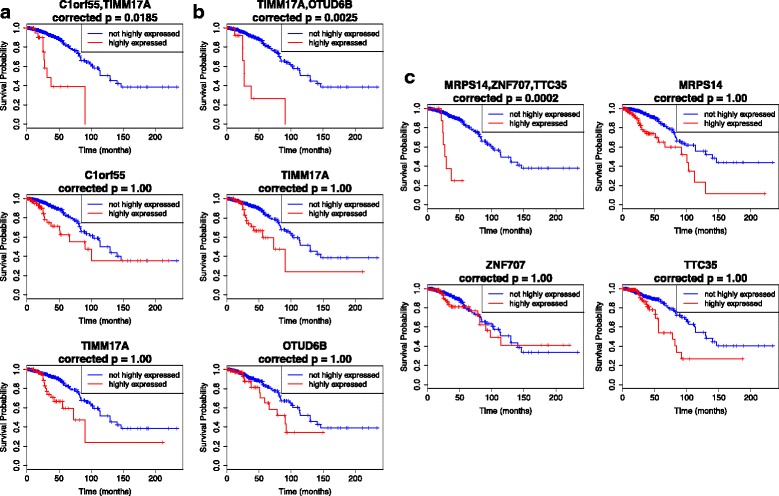
Kaplan-Meier plots for the top three markers in Table 2, and the corresponding KM plots for individual genes in combination markers. In the combinations, all genes involved are assumed to have high expressions. For all figures, the red curves represent the survival probability of individuals with highly expressed genes/gene combinations, while the blue curves represent the survival probability of individuals with non-highly expressed genes/gene combinations. Indicated *p*-values are the adjusted log-rank *p*-values using the total correction factor *k*=556284. If the adjusted *p*-values exceed 1.0, *p*=1.0 is used. **a** The 2-gene combination C1orf55,TIMM17A (uppermost) and the respective individual KM plots for C1orf55 (middle) and TIMM17A (lowermost); **b** The 2-gene combination TIMM17A,OTUD6B (uppermost) and the respective KM plots for the two genes in the combination; **c** The KM plot for the 3-gene combination MRPS14,ZNF707,TTC35 (top left), and the succeeding respective plots for the individual genes

**Fig. 3:**
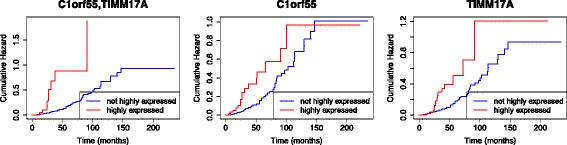
Cumulative hazard plots for the top combination marker C1orf55,TIMM17A (left-most) in Table 2, and the corresponding individual genes

To illustrate the effects of interactions vs single gene on patient survival, Kaplan-Meier plots of the first 3 gene combinations from Table 2 are given in Fig. 2. It is worth noting that the individual genes will never be statistically significant for *α*=0.05 and *k*=556284 (adjusted *p*-value is large so we set *p*=1.0 in the figures), but their combinations yield statistically significant results, e.g. C1orf55 and TIMM17A in Fig. 2a. Notable difference between the divergence patterns of the survival curves of combinations vs individual genes can be observed. Moreover, the impact of these combinations of highly expressed genes can potentially severely aggravate patient survival, with median survival time from as early as around 25 months. This is also supported by evidence of the cumulative hazard for the combination, such as that for C1orf55,TIMM17A rapidly increasing before reaching *t*=50 months, compared to the individual gene cumulative hazards, as shown in Fig. 3.

### Analysis of TCGA ovarian serous cystadenocarcinoma data

For the TCGA-OV data, we obtained a total of 5193 candidate combinations from the 79 sets, with average correction factor of 12962 per set and a total correction factor of *k*=920351. After correction on the raw *p*-values, 2849 combinations with size of at most 28, and where 1893 combinations are present in more than one sample, are retained. Top interactions with the most number of occurrences in the data samples are enumerated in Table 3.

**Table 3:** Significant markers with the most number of occurrences in the ovarian serous cystadenocarcinoma data

Gene combination	Adj. Log-rank *p*-value	No. of occurrences
GGCX	0.0061	33
MTF1,NBN	0.0068	13
ANTXR2	0.0029	12
GALNT10	0.0092	10
RTEL1,RTFDC1,CDK5RAP1	0.0115	10
KXD1,DDA1,C19orf43	0.0295	10

**Fig. 4:**
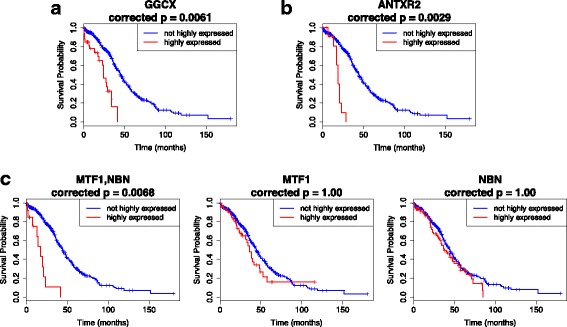
Kaplan-Meier plots for the top three markers in Table 3, and the corresponding KM plots for the individual genes in combination markers. In the combinations, all genes involved are assumed to have high expressions. For all figures, the red curves represent the survival probability of individuals with highly expressed genes/gene combinations, while the blue curves represent the survival probability of individuals with non-highly expressed genes/gene combinations. Indicated *p*-values are the adjusted log-rank *p*-values using the total correction factor *k*=920351. If the adjusted *p*-values exceed 1.0, *p*=1.0 is used. **a** The KM plot for the single gene GGCX, which is the top marker in Table 3 (most number of occurrences); **b** The KM plot for the single gene ANTXR2, the third marker with most number of occurrences; **c** The KM plot for the 2-gene combination MTF1,NBN (left-most), and the respective plots for the individual genes

**Fig. 5:**
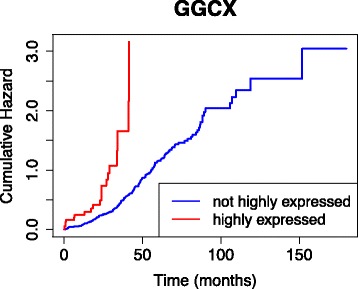
Cumulative hazard plot for GGCX, the top gene marker in Table 3

Similar to the TCGA-BRCA results, genes in the interactions potentially affecting the ovarian cancer survival include known oncogenes and novel candidates. As an example, high expressions of GGCX, the top gene in Table 3, has been observed in bladder cancer [24] and has been linked to a susceptibility locus in prostate cancer [25], encouraging subsequent studies of this gene and its role in cancer. Also, MTF1 and NBN genes in combination two have evidence of high gene expressions in lung, breast, and cervical cancer tumors [26], and mutations associated with cancer occurrence, such as breast, prostate and stomach cancers [27, 28], respectively. Further, studies suggest that the ANTXR2 (CMG2) gene plays a significant role in angiogenesis and promotes proliferation of endothelial cells and form and structure development during angiogenesis in cancers such as breast cancer [29, 30]. All of these imply potential effects of detected interactions in cancer risk and survival. Kaplan-Meier plots in Fig. 4 for the first three interactions in Table 3 also provide validation on the effects of these genes on patient prognosis, with effects of MTF1 and NBN genes significantly stronger when considering interactions, compared to individual effects (Fig. 4c). The rapid decrease in the survival curves corresponding to high expression of the markers are also noticeable, with median survival time also attained as early as two years. As expected, there is severe rapid increase in the cumulative force of mortality for such markers, which can be seen for the GGCX gene in Fig. 5.

### Validation of results

As further support to the resulting combinations detected by the proposed algorithm, we used separate breast and ovarian cancer data sets to check if these combinations are also statistically significant survival markers for these data. Gene expression and clinical data for breast and ovarian cancers were obtained from the National Center for Biotechnology Information Gene Expression Omnibus with accession numbers GSE2034 [31], GSE25066 [32] and GSE3494 [33] for breast cancer, and GSE13876 [34] and GSE49997 [35] for ovarian cancer. Raw values given in GSE2034 and GSE13876 were log2-transformed and then median-centered. On the other hand, data values given in the other data sets were already log-transformed and normalized, respectively (see [32, 33, 35]). Modified *z*-scores were computed using the normalized values, and two types of binarization were applied thereafter. The first one is similar to our experiment settings focusing on high expressions of the genes of interest included in the combinations: *z*-scores greater than 2 were set to 1, otherwise, set to 0. The second one considers the case when the genes of interest have low expressions, for which we set entries with *z*-scores less than − 0.5 to 1, otherwise, set to 0. Probes were mapped to genes using their Gene Entrez IDs, with some genes mapped to multiple probes in the data. Therefore, we checked all possible probe combinations of each respective gene combination, provided all genes in the combination have a corresponding probe in the data. Otherwise, such combinations cannot be assessed. Survival with various events of interest were analyzed using the validation data, namely: relapse with time to relapse or last follow-up (GSE2034), distant recurrence-free survival with time from operation to the first distant recurrence (GSE25066), disease-specific survival with DSS time (GSE3494), overall survival with time from primary surgery (GSE13876), and progression free survival (PFS) with survival time that the disease does not get worse during or post-surgery. The summary of proportions of statistically significant combinations found in the validation data sets, i.e., their raw *p*-value is less than 0.05, is given in Table 4. For combinations with multiple corresponding probe sets, the combination is statistically significant if at least one of the matching probe sets is statistically significant.

**Table 4:** Number of combinations that are also statistically significant using the validation data

(a) Validated BRCA results				
Expression	GSE2034	GSE25066	GSE3494-GPL96	GSE3494-GPL97
High	54/239 (22.59%)	37/172 (21.51%)	74/286 (25.87%)	23/106 (21.70%)
Low	466/2092 (22.28%)	404/2073 (19.49%)	421/2079 (20.25%)	105/670 (15.67%)
Total	509/2092 (24.33%)	428/2073 (20.65%)	485/2079 (23.33%)	123/670 (18.36%)
(b) Validated OV results				
Expression	GSE13876	GSE49997		
High	15/300 (5.00%)	15/108 (13.89%)		
Low	195/1526 (12.78%)	140/1444 (9.70%)		
Total	209/1526 (13.70%)	155/1444 (10.73%)		

Percentage values indicate the portion of statistically significant combinations from all combinations that can be matched in the data set. ‘High’ results use the binarization similar to our experiment settings where *z*-scores greater than 2 were set to 1, otherwise, 0. ‘Low’ results consider the case when the genes of interest are all lowly expressed and entries with *z*-scores less than − 0.5 are set to 1, otherwise, 0. All combinations were tested using these two binarizations, hence, the respective lists of statistically significant combinations found in the validation data may overlap. The ‘Total’ indicates the total number of unique combinations (high or low)that can be matched in the data and the portion of which are statistically significant

### Extensions and limitations of the model

An advantage of the algorithm presented here over other methods is its flexibility on the type of data used for analysis. It can easily deal with other genomic data such as SNPs or copy number variations provided values can be binarized. Moreover, scope of the application can be expanded to any type of disease (i.e., non-cancer diseases) and event of interest (e.g. cancer recurrence, remission, effectivity of treatment). The method can also be extended to continuous values, as techniques for significant pattern mining dealing with real-valued data have also been proposed [36].

While the proposed method can detect high order interactions without any theoretical limitations to the order of interaction, it is not without cost. One caveat of the algorithm is the calling of LCM multiple times, making it very time-consuming, especially for large-scale data, hence the data division performed in the analyses. A faster version for LAMP has been proposed [13], invoking the LCM algorithm only once with depth-first search, making it 10 to 100 times faster than the original. To utilize this approach, certain adjustments on the current algorithm must be applied.

Another shortcoming of the method is the relaxed minimum *p*-value bound, which returns very small *p*-values. This also causes the algorithm to run longer, due to the longer time it takes to terminate pruning in the LCM algorithm. The value of *λ* decreases unnecessarily, therefore increasing the number of testable items. While the correction factor is still significantly smaller than what would have been if Bonferroni correction is used, a tighter bound is still preferred.

## Conclusion

In this study, we presented a novel approach to finding potentially relevant high order gene markers that affect disease prognosis. By utilizing existing significant pattern mining techniques, our method can find multiple order combinations associated with the survival probabilities of affected and unaffected individuals while controlling the FWER and not being computationally expensive. Applying our algorithm to existing cancer survival study data yielded interactions involving genes already associated with cancer prognosis from existing literatures, as well us genes whose roles in cancer are still unknown.

## Abbreviations

FWER: Family-wise error rate
KM: Kaplan-Meier
LAMP: Limitless-arity multiple-testing procedure
LCM: Linear-time closed itemset miner
TCGA: The cancer genome atlas

## References

[1] Li J, Lenferink AE, Deng Y, Collins C, Cui Q, Purisima EO (2010). Identification of high-quality cancer prognostic markers and metastasis network modules. Nat Commun.

[2] Martinez-Ledesma E, Verhaak RG, Trevino V (2015). Identification of a multi-cancer gene expression biomarker for cancer clinical outcomes using a network-based algorithm. Sci Rep.

[3] Mehta S, Shelling A, Muthukaruppan A, Lasham A, Blenkiron C, Laking G, Print C (2010). Predictive and prognostic molecular markers for cancer medicine. Ther Adv Med Oncol.

[4] Suzuki K, Kachala SS, Kadota K, Shen R, Mo Q, Beer DG (2011). Prognostic immune markers in non-small cell lung cancer. Clin Cancer Res.

[5] Wang Z, Chen G, Wang Q, Lu W, Xu M (2017). Identification and validation of a prognostic 9-genes expression signature for gastric cancer. Oncotarget.

[6] van’t Veer LJ, Dai H, van de Vijver MJ, He YD, Hart AA, Mao M (2002). Gene expression profiling predicts clinical outcome of breast cancer. Nature.

[7] Terada A, Okada-Hatakeyama M, Tsuda K, Sese J (2013). Statistical significance of combinatorial regulations. Proc Natl Acad Sci USA.

[8] Uno T, Asai T, Uchida Y, Arimura H. (LCM): An efficient algorithm for enumerating frequent closed item sets In: Goethals B, MJ Z, editors. Proceedings of the ICDM 2003 Workshop on Frequent Itemset Mining Implementation: 2003.

[9] Tarone R (1990). A modified bonferroni method for discrete data. Biometrics.

[10] Mantel N, Haenszel W (1959). Statistical aspects of the analysis of data from retrospective studies of disease. J Natl Cancer Inst.

[11] duVerle DA, Takeuchi I, Murakami-Tonami Y, Kadomatsu K, Tsuda K (2013). Discovering combinatorial interactions in survival data. Bioinformatics.

[12] Bland JM, Altman DG (2004). The logrank test. BMJ.

[13] Calders T, Esposito F, Hüllermeier E, Meo R (2014). A Fast Method of Statistical Assessment for Combinatorial Hypotheses Based on Frequent Itemset Enumeration.

[14] Kuritz SJ, Landis JR, Koch GG (1988). A general overview of Mantel-Haenszel methods: applications and recent developments. Annu Rev Public Health.

[15] Network TCGA (2012). Comprehensive molecular portraits of human breast tumours. Nature.

[16] Network TCGAR (2011). Integrated genomic analyses of ovarian carcinoma. Nature.

[17] Xu X, Qiao M, Zhang Y, Jiang Y, Wei P, Yao J (2010). Quantitative proteomics study of breast cancer cell lines isolated from a single patient: discovery of TIMM17A as a marker for breast cancer. Proteomics.

[18] Salhab M, Patani N, Jiang W, Mokbel K (2012). High TIMM17A expression is associated with adverse pathological and clinical outcomes in human breast cancer. Breast Cancer.

[19] Yang X, Si Y, Tao T, Martin TA, Cheng S, Yu H (2016). The Impact of TIMM17A on Aggressiveness of Human Breast Cancer Cells. Anticancer Res.

[20] Jo U, Cai W, Wang J, Kwon Y, D’Andrea AD, Kim H (2016). PCNA-Dependent Cleavage and Degradation of SDE2 Regulates Response to Replication Stress. PLoS Genet.

[21] Gaillard H, Garcia-Muse T, Aguilera A (2015). Replication stress and cancer. Nat Rev Cancer.

[22] Sobol A, Askonas C, Alani S, Weber MJ, Ananthanarayanan V, Osipo C, Bocchetta M (2017). Deubiquitinase OTUD6B Isoforms Are Important Regulators of Growth and Proliferation. Mol Cancer Res.

[23] Sircoulomb F, Nicolas N, Ferrari A, Finetti P, Bekhouche I, Rousselet E (2011). ZNF703 gene amplification at 8p12 specifies luminal B breast cancer. EMBO Mol Med.

[24] Cheng S, Andrew AS, Andrews PC, Moore JH (2016). Complex systems analysis of bladder cancer susceptibility reveals a role for decarboxylase activity in two genome-wide association studies. BioData Min.

[25] Kote-Jarai Z, Olama AA, Giles GG, Severi G, Schleutker J, Weischer M (2011). Seven prostate cancer susceptibility loci identified by a multi-stage genome-wide association study. Nat Genet.

[26] Shi Y, Amin K, Sato BG, Samuelsson SJ, Sambucetti L, Haroon ZA (2010). The metal-responsive transcription factor-1 protein is elevated in human tumors. Cancer Biol Ther.

[27] Seemanova E, Jarolim P, Seeman P, Varon R, Digweed M, Swift M, Sperling K (2007). Cancer risk of heterozygotes with the NBN founder mutation. J Natl Cancer Inst.

[28] Uzunoglu H, Korak T, Ergul E, Uren N, Sazci A, Utkan NZ (2016). Association of the nibrin gene (NBN) variants with breast cancer. Biomed Rep.

[29] Reeves CV, Dufraine J, Young JA, Kitajewski J (2010). Anthrax toxin receptor 2 is expressed in murine and tumor vasculature and functions in endothelial proliferation and morphogenesis. Oncogene.

[30] Ye L, Sun PH, Sanders AJ, Martin TA, Lane J, Mason MD, Jiang WG (2014). Therapeutic potential of capillary morphogenesis gene 2 extracellular vWA domain in tumour-related angiogenesis. Int J Oncol.

[31] Wang Y, Klijn JG, Zhang Y, Sieuwerts AM, Look MP, Yang F, Talantov D, Timmermans M, Meijer-van Gelder ME, Yu J, Jatkoe T, Berns EM, Atkins D, Foekens JA (2005). Gene-expression profiles to predict distant metastasis of lymph-node-negative primary breast cancer. Lancet.

[32] Itoh M, Iwamoto T, Matsuoka J, Nogami T, Motoki T, Shien T, Taira N, Niikura N, Hayashi N, Ohtani S, Higaki K, Fujiwara T, Doihara H, Symmans WF, Pusztai L (2014). Estrogen receptor (ER) mRNA expression and molecular subtype distribution in ER-negative/progesterone receptor-positive breast cancers. Breast Cancer Res Treat.

[33] Miller LD, Smeds J, George J, Vega VB, Vergara L, Ploner A, Pawitan Y, Hall P, Klaar S, Liu ET, Bergh J (2005). An expression signature for p53 status in human breast cancer predicts mutation status, transcriptional effects, and patient survival. Proc Natl Acad Sci USA.

[34] Crijns AP, Fehrmann RS, de Jong S, Gerbens F, Meersma GJ, Klip HG, Hollema H, Hofstra RM, te Meerman GJ, de Vries EG, van der Zee AG (2009). Survival-related profile, pathways, and transcription factors in ovarian cancer. PLoS Med.

[35] Pils D, Hager G, Tong D, Aust S, Heinze G, Kohl M, Schuster E, Wolf A, Sehouli J, Braicu I, Vergote I, Cadron I, Mahner S, Hofstetter G, Speiser P, Zeillinger R (2012). Validating the impact of a molecular subtype in ovarian cancer on outcomes: a study of the OVCAD Consortium. Cancer Sci.

[36] Sugiyama M, Borgwardt KM. Finding Significant Combinations of Continuous Features. arXiv preprint arXiv:1702.08694. 2017. https://arxiv.org/abs/1702.08694.

